# Multi-omics reveals age-related differences in the diaphragm response to mechanical ventilation: a pilot study

**DOI:** 10.1186/s13395-021-00267-4

**Published:** 2021-05-03

**Authors:** Qiong Lyu, Ya Wen, Xiang Zhang, Alex B. Addinsall, Nicola Cacciani, Lars Larsson

**Affiliations:** 1 Department of Physiology and Pharmacology, Karolinska Institutet, Bioclinicum, J8:30, SE-171 77 Stockholm, Sweden; 2 Department of General Practice, The First Affiliated Hospital of Chongqing Medical University, 400016 Chongqing, China; 3 Department of Clinical Neuroscience, Karolinska Institutet, Bioclinicum, J8:30, SE-171 77 Stockholm, Sweden

**Keywords:** Mechanical ventilation, Age, VIDD, Hyperinflammation, COVID-19

## Abstract

**Background:**

Old age is associated with a significantly increased mortality in COVID-19 patients exposed to long-term controlled mechanical ventilation (CMV) and suggested to be due to the hyperinflammatory response associated with the viral infection. However, our understanding of age-related differences in the response to CMV in the absence of a viral infection remains insufficient.

**Methods:**

Young (7–8 months) and old (28–32 months) F344 BN hybrid rats were exposed to the ICU condition for 5 days, i.e., complete immobilization, mechanical ventilation, and extensive monitoring. Transcriptomic (RNA-Seq) and proteomics (Proximity Extension Assay) analyses of the diaphragm and proteomics analysis of plasma were conducted to investigate the molecular differences between young and old rats exposed to the ICU condition.

**Results:**

According to multi-omics analyses, significant differences were observed in the diaphragm between young and old rats in response to 5 days CMV and immobilization. In young rats, metabolic pathways were primarily downregulated in response to immobilization (post-synaptic blockade of neuromuscular transmission). In old rats, on the other hand, dramatic immune and inflammatory responses were observed, i.e., an upregulation of specific related pathways such as “IL-17 signaling pathway”, along with a higher level of inflammatory factors and cytokine/chemokine in plasma.

**Conclusions:**

The dramatically increased mortality in old ICU patients with COVID-19-associated hyperinflammation and cytokine storm need not only reflect the viral infection but may also be associated with the ventilator induced diaphragm dysfunction (VIDD) and hyperinflammatory responses induced by long-term CMV per se. Although mechanical ventilation is a life-saving intervention in COVID-19 ICU patients, CMV should be cautiously used especially in old age and other means of respiratory support may be considered, such as negative pressure ventilation.

**Supplementary Information:**

The online version contains supplementary material available at 10.1186/s13395-021-00267-4.

## Background

The novel coronavirus SARS-CoV-2 causes respiratory disease (COVID-19) which is more severe in old patients, frequently requiring intensive care and long-term mechanical ventilation (MV) [1]. The morbidity/mortality rate is higher in old patients with COVID-19 [2] and the hyperinflammatory response induced by SARS-CoV-2 has been forwarded as the major pathological process leading to disease severity and death in infected older patients [3–5]*.* ‘Inflammaging’ is the term used to describe this chronic low-grade systemic inflammation accompanying aging which predicts susceptibility to age-related pathologies [5]. However, a key unknown is the relationship between inflammaging and the hyperinflammation that occurs in older COVID-19 patients with severe disease vs. consequences of the intensive care unit (ICU) condition per se.

MV is a life-saving intervention in patients with acute respiratory failure and frequently used for long durations in COVID-19 patients [1, 6]. However, MV is also associated with negative consequences in the respiratory system, such as ventilator-induced lung injury (VILI) [7] and the ventilator-induced diaphragm dysfunction (VIDD) [8]. The rapid decrease in diaphragm muscle endurance and strength associated with VIDD induces extubation failure, prolonged weaning from ventilator with increased duration of ICU stay and staggering negative effects on ICU mortality and healthcare expenditures, with old patients being at higher risk. This being a major concern in the modern era of critical care during the current COVID-19 pandemic [9]. It is hypothesized that MV, CMV in particular, induces VILI and the release of factors affecting peripheral organs including diaphragm muscle, contributing to the pathogenesis of VIDD. In the experimental ICU model used in this study, all rats develop VILI and VIDD in response to long-term CMV [10], with old rats being more severely affected than young [11]. Notably, it is also reported that old patients with COVID-19 may benefit less from MV compared to those who are younger and healthier at baseline [12].

The current study was therefore undertaken to compare CMV-induced molecular alterations in diaphragm and plasma between young and old rats exposed to 5 days CMV, using a unique experimental ICU model. This model replicates both genotypic and phenotypic features of the compromised skeletal muscle structure and function observed clinically in patients with VIDD [10, 13–16]. Compared to young rats, we observed dramatic inflammatory activities in the diaphragm, contributing to more severe VIDD, and a higher systemic inflammation level in old rats. Our study suggests that, besides the viral infection, the age-related response to the CMV *per se* also contributes to hyperinflammation and cytokine storm reported in old patients with COVID-19 responsible for the increased mortality in old age.

## Materials and methods

### Animals and samples

A total of 10 female F344 brown Norway hybrid rats at the age of 7–8 months (young, *n* = 6) and 28–32 months (old, *n* = 4) were obtained from the National Institute of Aging (NIA, Bethesda, MD). Three young and 2 old rats were randomly allocated into 0-day control or 5-day CMV group, respectively. The rats were anesthetized, pharmacologically paralyzed by post-synaptic blockade of the neuromuscular transmission (neuromuscular blocker (NMB); α-cobra-toxin), mechanically ventilated and monitored extensively 24 h/day for 5 days and compared with 0-day controls. Animals were maintained in caloric, protein, and fluid balance, including intra-arterial and intra-venous solution. The 0-day control animals were anesthetized, spontaneously breathing, given intra-arterial and intra-venous solutions, and sacrificed within 1 h after the initial anesthesia and surgery. The 5-day experimental animals were ventilated through a per os coaxial tracheal cannula at 72 breaths/min with an inspiratory and expiratory ratio of 1:2 and a minute volume of 180–200 ml and gas concentrations of 40% O_2_, 57% N_2_, and 3% CO_2_, delivered by a precision (volume drift < 1%/week) volumetric respirator. Intermittent hyperinflations (6 per hour at 20 cm H_2_O) and constant positive end-expiratory pressure (PEEP; 1.5 cm H_2_O) for 3 s, were supplied in order to prevent lungs derecruitment and atelectasis. NMB was induced on the first day (150 μg IA α-cobra-toxin) and maintained by continuous IA infusion (250 μg/day). Invasive mechanical ventilation started after the NMB induction, at the first clinical signs of diaphragm paralysis, carefully avoiding episodes of oxygen desaturation. Experimental animals were monitored for heart rate, electrocardiogram (ECG), electroencephalogram (EEG), arterial and venous blood pressure, pulsoxymetry, body temperature, respiratory cycle pressures, end-tidal CO_2_ (Et CO_2_), and urine output (for details see previous publication [17]). During the 5 days CMV, in no case did animals show any signs of infections and septicemia. The diaphragm muscle and plasma were collected immediately after euthanasia.

### Library preparation and transcriptome sequencing

RNA was extracted using RNeasy® Fibrous tissue mini kit (Qiagen, Inc., Valencia, CA, USA) according to the manufacturer’s instruction. After quality control, a total amount of 1 μg RNA per sample was used as input material for library preparations and barcoded using NEBNext® Ultra^TM^ RNA Library Prep Kit for Illumina® (NEB, USA). The library preparations were subject to sequencing on an Illumina platform (HiSeq Xten).

### Olink proteomics assay

Diaphragm and plasma proteins from each sample were lysed in T-PER lysis buffer (Thermo Fisher Scientific) with complete protease inhibitors (Sigma-Aldrich) and diluted to 1 ng/μl. Proteins lysates were examined by the Olink Mouse Exploratory Panel (Olink Proteomics AB, Uppsala, Sweden) according to the manufacturer’s instructions. In brief, pairs of oligonucleotide-labeled antibody probes are incubated with the sample. Once bound to their targeted protein the oligonucleotides probes that are in close proximity hybridize in a pair-wise manner. The addition of a DNA polymerase leads to proximity-dependent DNA polymerization, generating a unique PCR target sequence, which is subsequently detected using a microfluidic real-time PCR instrument. The quantification cycle (Cq) values from a DNA extension control are subtracted from the measured Cq value and an interplate correction factor applied to yield a normalized protein expression value (NPX), which is log2-transformed. All assay validation data are available on the manufacturer’s website (www.olink.com).

### Differential expression analysis

After quality control, the paired-end clean reads were mapped to the rat Ensemble reference genome using HISAT2 [18]. HTSeq [19] was used to count the read numbers mapped to each gene. Subsequently, differential expression analysis was performed using DESeq2 [20]. Genes with an adjusted *p* value < 0.05 and absolute fold change > 2 were assigned as differentially expressed genes (DEGs).

Prior to analysis, the protein level from Olink proteomics platform was converted into a linear scale (2^NPX^). Generalized linear regression was used for the identification of differentially expressed proteins (DEPs). Proteins with an adjusted *p* value < 0.05 and absolute fold change > 2 were defined as differentially expressed.

### Enrichment analyses

To extract biological meaning from a list of DEGs and DEPs, they were subject to Gene Ontology (GO) and KEGG pathway enrichment analysis by ClusterProfiler [21]. The terms from GO and KEGG enrichment analyses were considered significantly enriched when adjusted *p* value was less than 0.05.

Fold change information of each DEGs was added to our enrichment analyses, and reflected as a *z*-score [22, 23]. *Z*-score is a calculation to predict of the status of the enriched KEGG pathway. *Z*-score is calculated as

$$ \mathrm{zscore}=\frac{\left(\mathrm{up}-\mathrm{down}\right)}{\sqrt{\mathrm{count}}} $$

*Count* is the number of genes assigned to an enriched term. Whereas *up* and *down* are the number of assigned genes upregulated in the data or downregulated, respectively.

## Results

### Gene and protein signatures in the diaphragm and the plasma in response to CMV

**Fig. 1:**
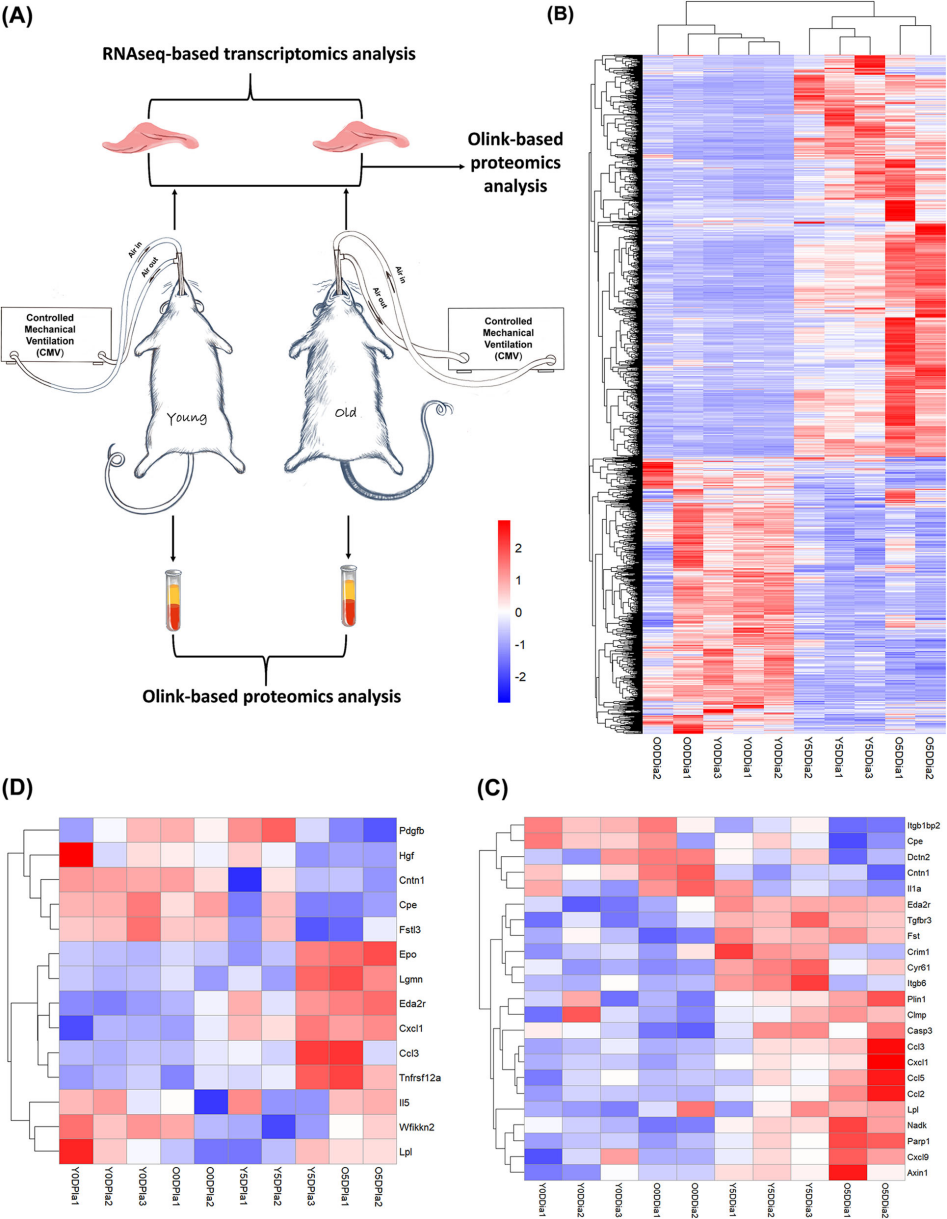
Hierarchical clustering analysis of DEGs and DEPs identified in the diaphragm and plasma in response to CMV. **a** Schematic of experimental design. **b** Heatmap of DEGs and **c** Heatmap of DEPs in the diaphragm among 0-day controls and 5-day CMV groups in both young and old rats. **d** Heatmap of DEPs in the plasma among 0-day controls and 5-day CMV groups in both young and old rats. CMV: controlled mechanical ventilation; DEGs: differentially expressed genes; DEPs: differentially expressed proteins. Y0DDia (Y5DDia) represents diaphragm sample of young rat from 0-day control (5-day CMV); Y0DPla (Y5Dpla) represents plasma sample of young rat from 0-day control (5-day CMV); O0DDia (O5DDia) represents diaphragm sample of old rat from 0-day control (5-day CMV); O0DPla (O5Dpla) represents plasma sample of old rat from 0-day control (5-day CMV)

The experimental schematics are illustrated in Fig. 1a. The diaphragm was subject to RNA-seq and proteomic analyses, and plasma to proteomic analysis.

According to RNA-seq analysis, a total of 12,321 genes were detected (screened by a default threshold of Fragments Per Kilobase Million, FPKM value set as (1) among young and old rats from 0-day control groups and 5-day CMV groups (Supplemental Figure 1A). In young rats, a total of 2163 differentially expressed genes (DEGs; *p* value < 0.05 and absolute fold change > 2; see details in Supplemental Table 1A) were identified and the majority were downregulated after 5 days CMV (60% or 1295 genes). In old rats, on the other hand, a total of 1752 DEGs were identified, and the majority were upregulated (82% or 1445 genes) (Supplemental Figure 1B; see details in Supplemental Table 1B). Hierarchical clustering analysis of these CMV-induced DEGs in the diaphragm is shown in Fig. 1b. The clustering pattern demonstrates a distinct difference between 5-day CMV groups. Moreover, the clustering pattern also demonstrates the difference between old and young rats after but not before ventilation. This result suggests specific age-related molecular alterations in response to 5 days CMV and immobilization.

According to diaphragm proteomics analysis, a total of 8 and 19 differentially expressed proteins (DEPs; *p* value < 0.05 and absolute fold change > 2; see details in Supplemental Table 1C and D) were identified in response to 5 days CMV in young and old rats, respectively. Four of these DEPs were co-detected, including Itgb1bp2, Fst, Cyr61, and Eda2r. The detailed information (fold change and *p* value) of these DEPs is shown in Supplemental Figure 1C and D. The heatmap of these CMV-induced DEPs of the diaphragm is shown in Fig. 1c**.**

According to plasma proteomics analysis, a total of 10 DEPs and 9 DEPs (see details in Supplemental Table 2A and B) were identified in response to CMV in young and old rats, respectively. Five of these DEPs were co-detected, including Eda2r, Cxcl1, Ccl3, and Tnfrsf12a, and Cpe. The detailed information (fold change and *p* value) of these DEPs is shown in Supplemental Figure 1E and F. The heatmap of these CMV-induced DEPs of the plasma is shown in Fig. 1d.

### Age-related molecular differences in the diaphragm in response to CMV

#### GO enrichment analyses of DEGs and DEPs in the diaphragm

**Fig. 2:**
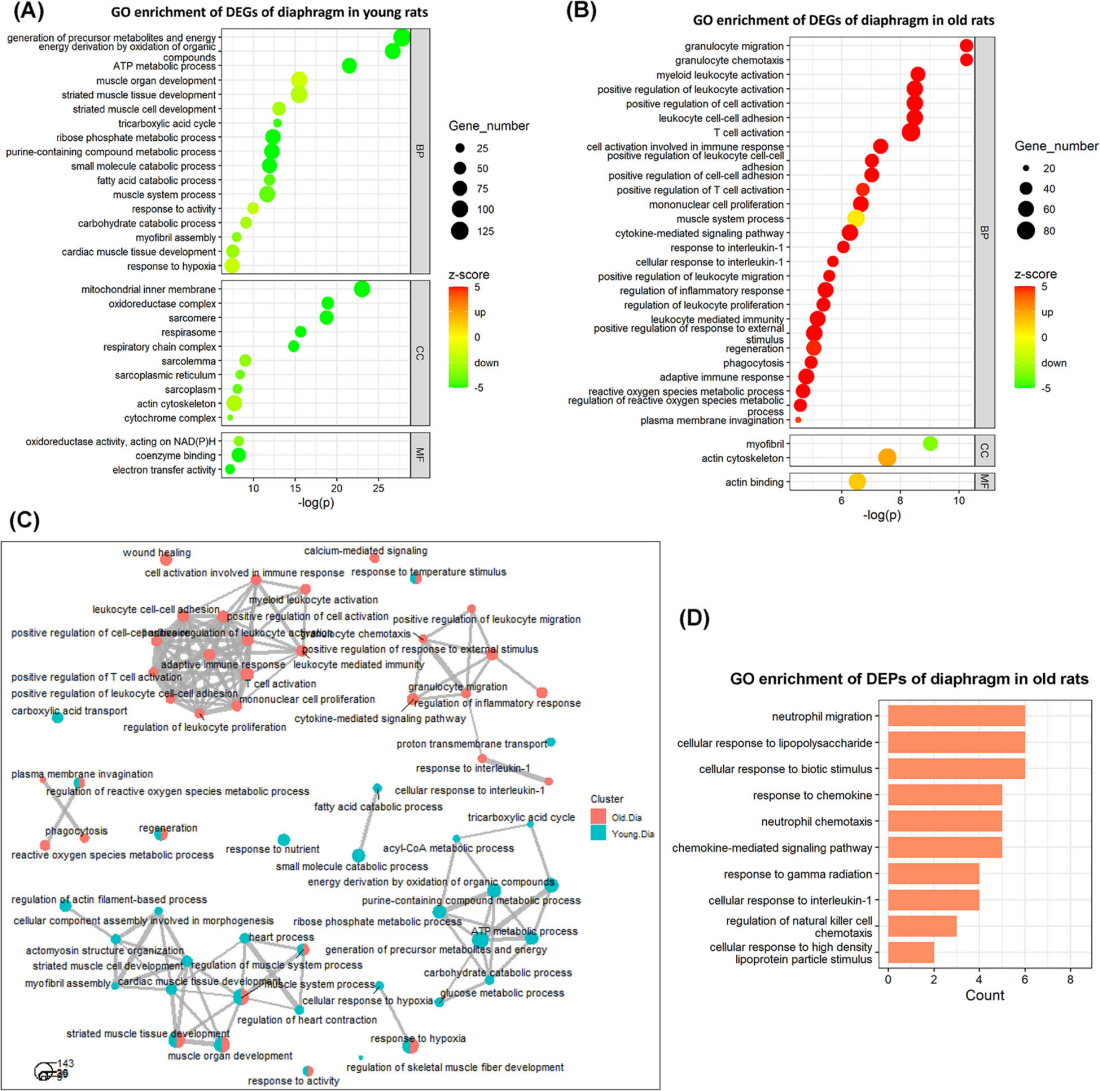
GO enrichment analyses of DEGs and DEPs of the diaphragm in response to CMV. The 30 top-ranked GO terms enriched by the DEGs of the diaphragm in **a** young rats and **b** old rats. **c** Comparative network analyses of the GO terms of both young and old rats. **d** The 10 top-ranked GO terms enriched by the DEPs of the diaphragm in old rats. BP: biological process; CC: cellular component; MF: molecular function. *Z*-score is used as a hint or a prediction of the status of the enriched GO terms. Negative *z*-score (in green) represents “downregulation” status, whereas positive *z*-score (in red) represents “upregulation” status of the GO terms

GO terms are divided into three sub-ontologies: biological process (BP), molecular function (MF), and cellular component (CC). In young rats, the 30 top-ranked GO terms consisting of 17 BP, 10 CC, and 3 MF category are shown in Fig. 2a (see more GO terms in Supplemental Table 3A). The negative *z*-scores of the majority GO terms indicate that the corresponding involved DEGs were primarily downregulated. For instances, the top 3 GO terms in BP category with very low *z*-score, including “generation of precursor metabolites and energy” (*z*-score − 8.89), “energy derivation by oxidation of organic compounds” (*z*-score − 8.05), and “ATP metabolic process” (*z*-score − 6.76), were dominated by large amounts of downregulated DEGs, suggesting a decrease in biological processes highly related to energy metabolism and production in the diaphragm. In addition, the top 3 GO terms in CC category, including “mitochondrial inner membrane” (*z*-score − 8.69), “oxidoreductase complex” (*z*-score − 6.38), and “sarcomere” (*z*-score − 5.18), indicate that many genes with significant downregulated expression encode not only mitochondrial components but also sarcomere components. The detailed fold change information of DEGs contained in these top-ranked GO terms is shown in Supplemental Figure 2.

In old rats, the 30 top-ranked GO terms containing 27 BP, 2 CC, and 1 MF categories are shown in Fig. 2b (see more GO terms in Supplemental Table 3B). The positive *z*-scores of the majority GO terms indicate that the corresponding DEGs were upregulated. For instance, the top 3 GO terms in the BP category with very high *z*-score included “granulocyte migration” (*z*-score 6.33), “granulocyte chemotaxis” (*z*-score: 6.00) and “myeloid leukocyte activation” (*z*-score 6.73), were dominated by large amounts of upregulated DEGs, suggesting a strong increase in biological processes related to immune cell activities in the diaphragm of old rats. Interestingly, the *z*-score of the top GO term in the CC category “myofibril (*z*-score − 3.54)” was negative, indicating significant downregulated expression of genes encoding myofibrillar components. Detailed fold change information of DEGs contained in these top-ranked GO terms is shown in Supplemental Figure 3.

Relationships among the mainly involved biological processes in the diaphragm of young and old rats in response to CMV are shown in Fig. 2c. The distinct clustering among young and old rats supports strong age-related differences in response to 5 days CMV. In young rats, relevant GO terms in BP category formed two clusters. One cluster suggests these biological processes influenced by CMV were mainly related to “generation of precursor metabolites and energy”. Another cluster suggests these biological processes influenced by CMV were mainly related to “muscle system processes.” In old rats, the many relevant GO terms in the BP category also formed two clusters. Both suggest these biological processes influenced by CMV were mainly related to immune and inflammation responses.

Furthermore, Fig. 2d shows the GO terms enriched by CMV-induced DEPs in the diaphragm in old rats (see more GO terms in Supplemental Table 3C and D). In young rats, there were no significantly enriched GO terms, which may be due to the small number of detected DEPs in the diaphragm of young rats. In old rats, on the other hand, multiple GO terms in BP category highly related to the chemokine-mediated activities were identified, such as the top 3 GO terms “chemokine-mediated signaling pathway,” “response to chemokine,” and “cellular response to chemokine,” supporting the above GO enrichment analysis of DEGs since chemokines play an important role in controlling immune system migration and positioning of immune cells in tissues.

#### KEGG pathways enrichment analyses of DEGs and DEPs in the diaphragm

**Fig. 3:**
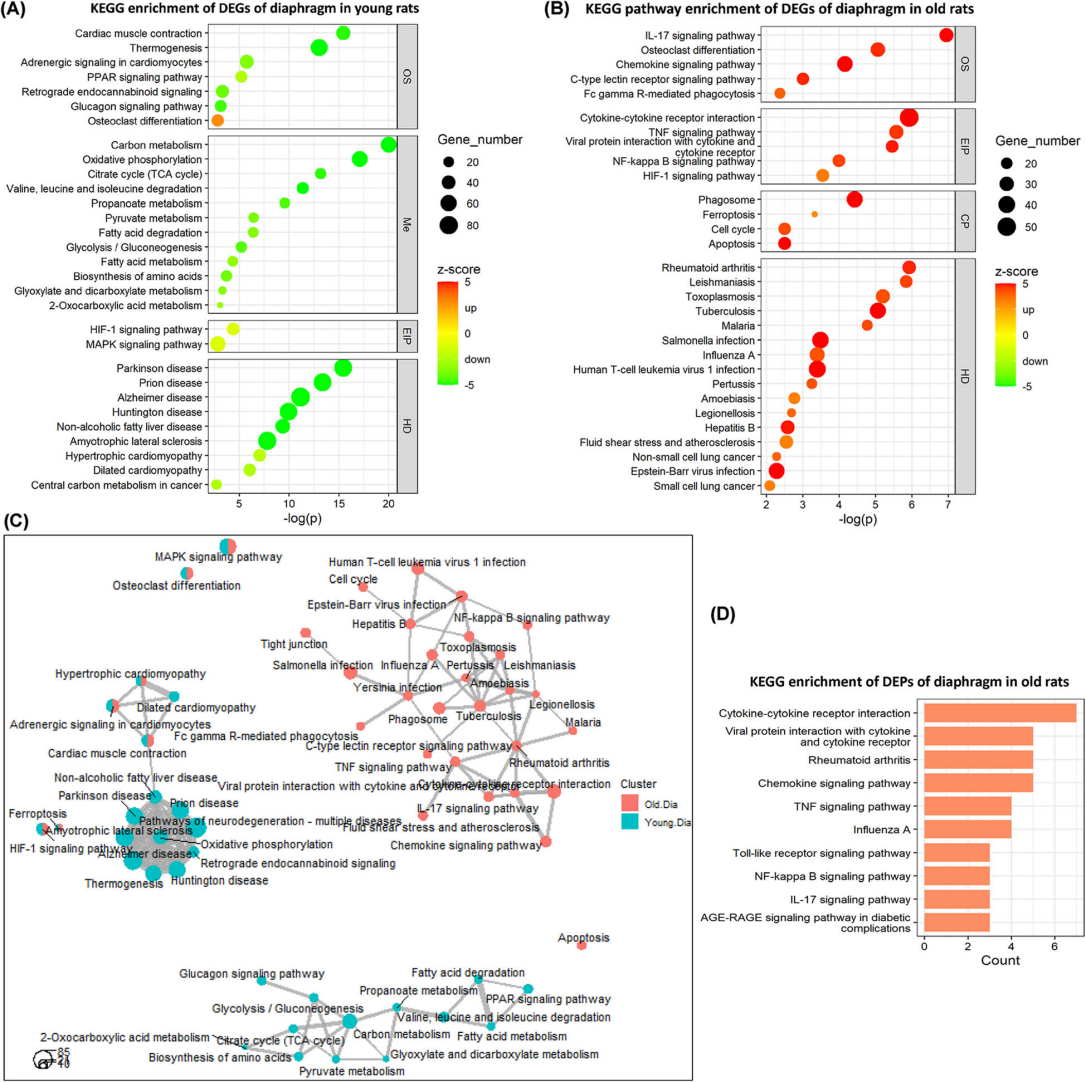
KEGG pathway enrichment analyses of DEGs and DEPs of the diaphragm in response to CMV. The 30 top-ranked KEGG pathways enriched by the DEGs of the diaphragm in **a** young rats and **b** old rats. **c** Comparative network analyses of the KEGG pathways of both young and old rats. **d** The 10 top-ranked KEGG pathways enriched by the DEPs of the diaphragm in old rats. OS: organismal system; Me: metabolism; EIP: environmental information processing; CP: cellular processes; HD: human diseases. *Z*-score is used as a hint or a prediction of the status of the enriched KEGG pathways. Negative *z*-score (in green) represents “inhibited” status, whereas positive *z*-score (in red) represents “activated” status of the KEGG pathways

KEGG pathways are officially classified into seven categories: “metabolism (Me),” “genetic information processing (GIP),” “environmental information processing (EIP),” “cellular processes (CP),” “organismal system (OS),” “human diseases (HD),” “drug development (DD).” In young rats, the 30 top-ranked KEGG pathways containing 7 OS, 12 Me, 2 EIP, and 9 HD categories are shown in Fig. 3a (see more KEGG pathways in Supplemental Table 4A). The majority of the KEGG pathways with very low *z*-scores indicate that the corresponding involved DEGs were mostly downregulated, such as “carbon metabolism” (Me; *z*-score − 6.76), “oxidative phosphorylation” (Me; *z*-score − 7.22), “citrate cycle (TCA cycle)” (Me; *z*-score − 4.80), and “calcium signaling pathway” (EIP; *z*-score − 3.65) (not the 30 top-ranked), suggesting inhibition of these pathways. For example, the inhibited status of KEGG pathway “oxidative phosphorylation” and “calcium signaling pathway” is illustrated in Supplemental Figure 4 and 5.

In old rats, the 30 top-ranked KEGG pathways consisting of 5 OS, 5 EIP and 4 CP, and 16 HD categories are shown in Fig. 3b (see more KEGG pathways in Supplemental Table 4B). The majority of KEGG pathways with very high *z*-scores indicate that the corresponding DEGs were mostly upregulated, such as “IL-17 signaling pathway” (OS; *z*-score 5.39), “chemokine signaling pathway” (OS; *z*-score 5.24), “cytokine-cytokine receptor interaction” (EIP; *z*-score: 6.38), “TNF signaling pathway” (EIP; *z*-score 4.64), “NF-kappa B signaling pathway” (EIP; *z*-score: 4.49), “phagosome” (CP; *z*-score 5.84), and “apoptosis” (CP; *z*-score 5.00), suggesting activation of these pathways.

The relationships among the KEGG pathways primarily involved in the diaphragm of young and old rats in response to CMV are shown in Fig. 3c. The distinct clusters among young and old rats in response to five days CMV demonstrate age-specific differences. Specifically, in young rats, there are many relevant KEGG pathways forming two clusters, which suggest these pathways influenced by CMV are mainly related to “carbon metabolism” and “oxidative phosphorylation.” In old rats, there are many relevant KEGG pathways forming one big cluster mainly related to inflammation and immune responses.

**Fig. 4:**
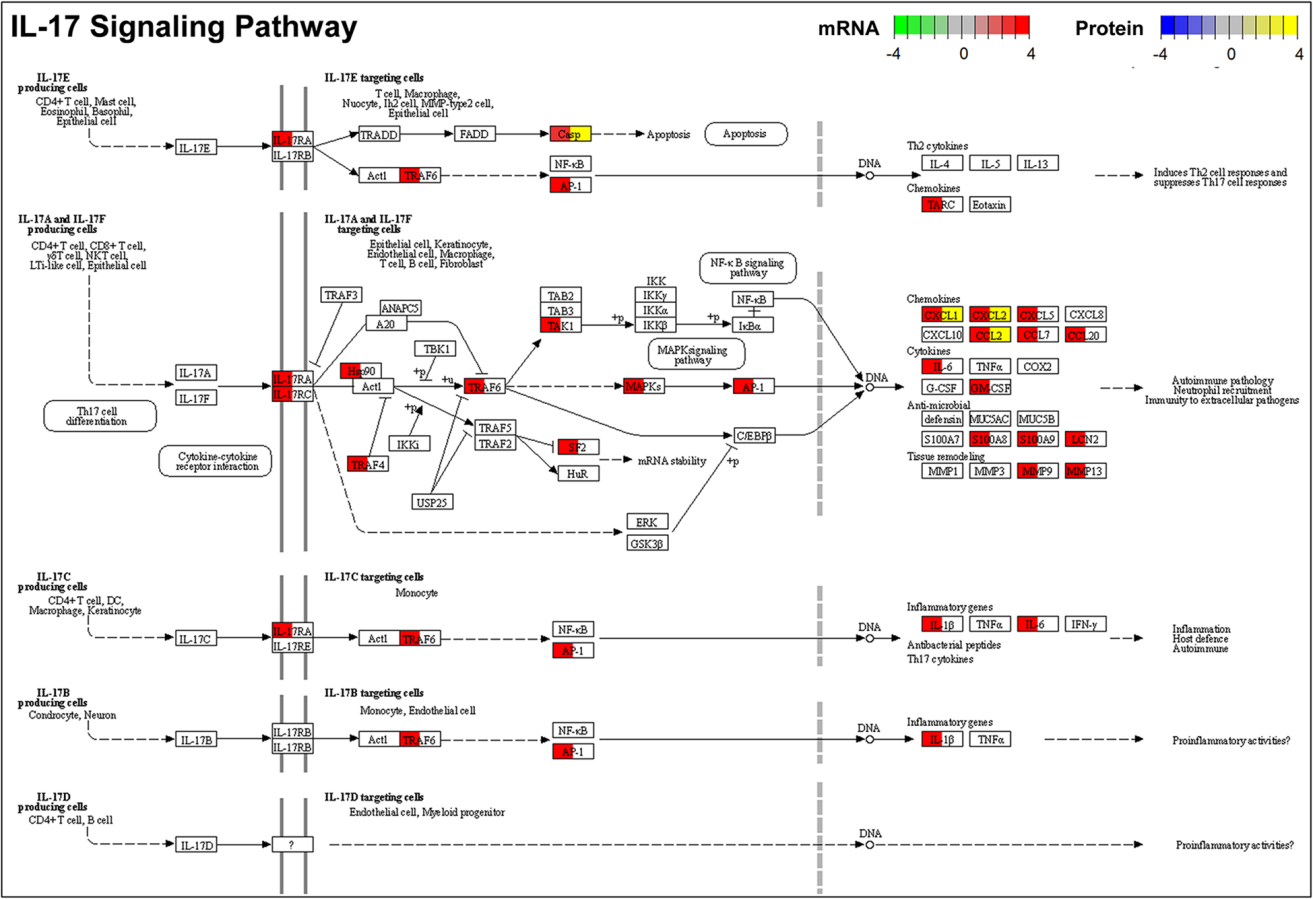
“IL-17 signaling pathway” and involved DEGs and DEPs of the diaphragm in response to CMV in old rats. Casp, Cxcl1, Cxcl2, and Ccl2 were identified both as the CMV-induced DEGs and CMV-induced DEPs. Fold change information of each DEG is indicated in color from low (green) to high (red). Fold change information of each DEP is indicated in color from low (blue) to high (yellow)

Furthermore, Fig. 3d shows the KEGG terms enriched by CMV-induced DEPs of the diaphragm in old rats (see more KEGG pathways in Supplemental Table 4C and D). In young rats, there were no significantly enriched KEGG pathways, which may be due to the small number of detected DEPs in the diaphragm of young rats. In old rats, the DEPs and their enriched KEGG pathways are consistent with the DEGs and their enriched pathways, such as “cytokine-cytokine receptor interaction” (Supplemental Figure 6), “TNF signaling pathway” (Supplemental Figure 7), “IL-17 signaling pathway” (Fig. 4), and “NF-kappa B signaling pathway” (Supplemental Figure 8).

### CMV induces a higher level of circulatory inflammatory factors in old age

**Fig. 5:**
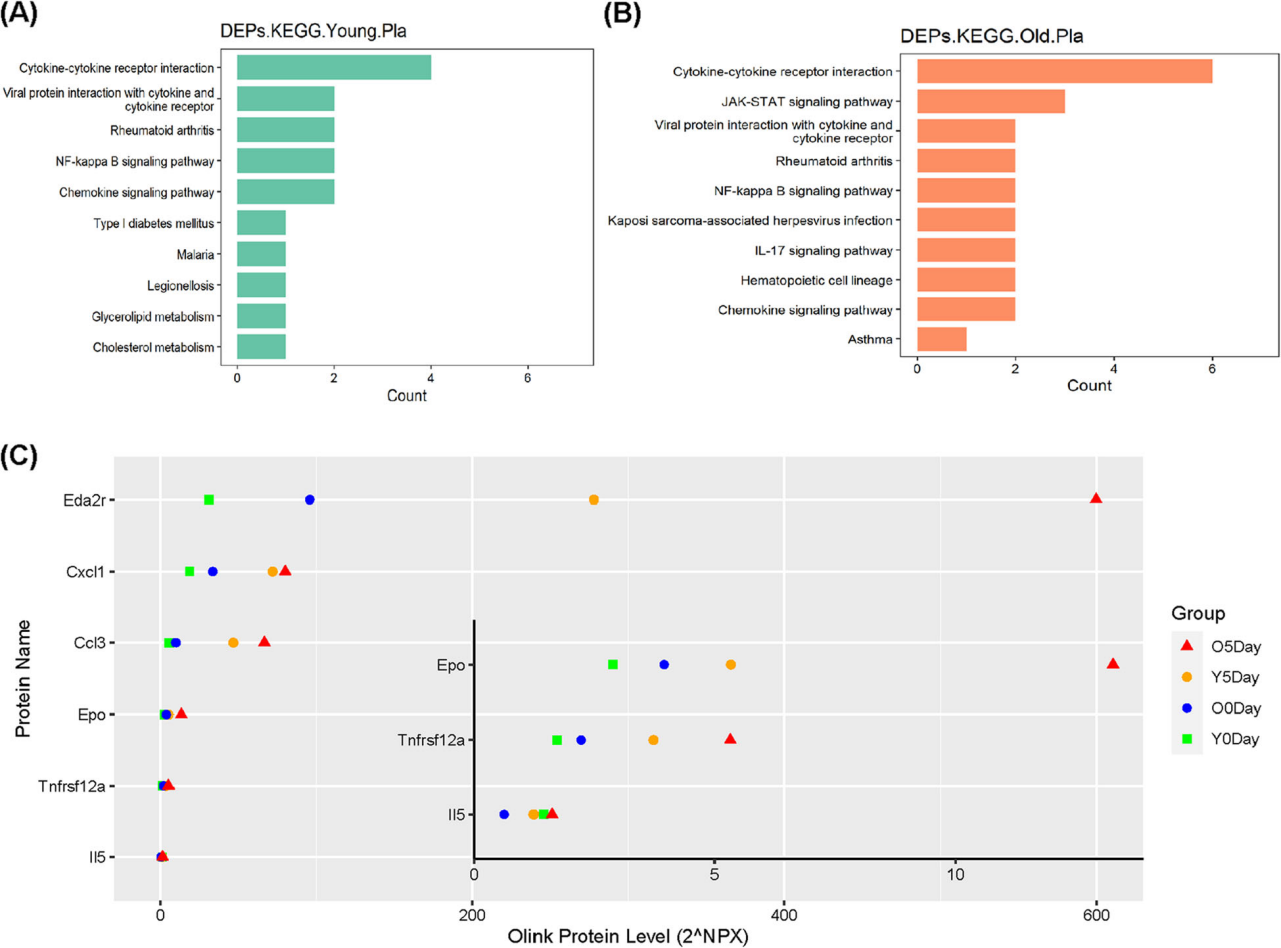
Functional enrichment analyses of DEPs of the plasma in response to CMV. KEGG pathway enrichment of CMV-induced DEPs of the plasma in **a** young rats and **b** old rats. **c** Olink protein levels of DEPs involved in the top KEGG pathways

KEGG pathways enriched by plasma DEPs demonstrate the main biological functions and pathways influenced by CMV. As Fig. 5a, b shows, there are many overlaps in the enriched KEGG pathways in young and old rats, such as “cytokine-cytokine receptor interaction,” “NF-kappa B signaling pathway,” “chemokine signaling pathway,” which suggest CMV may have a similar impact on the biological functions mainly related to inflammation and immune activities regardless of age (see more in Supplemental Table 5A and B).

Figure 5c shows the DEPs involved in the above inflammation relevant KEGG pathways, including “NF-kappa B signaling pathway,” “IL-17 signaling pathway,” “chemokine signaling pathway,” “cytokine-cytokine receptor interaction,” Dramatic increases in the protein levels of Eda2r, Cxcl1, Ccl3, EPO, and Tnfrsf12a were observed after 5 days CMV in both young and old rats. Interestingly, the basal protein levels of five DEPs (Eda2r, Cxcl1, Ccl3, Epo, and Tnfsf12a) were higher in old compared with young rats. The differences in the plasma protein levels of four of these five factors (Eda2r, Ccl3, Epo, and Tnfrsf12a) between young and old rats were enlarged after five days CMV.

## Discussion

The current experiments provide novel insights into age-related molecular alterations in the diaphragm in response to long-term CMV which ultimately may assist in the development of personalized countermeasures tailored to patients’ age. The pathophysiology and mechanisms underlying VIDD progression are complex and strongly related to age with negative consequences for mortality and morbidity. RNA-Seq transcriptomics and Olink proteomics provide effective means for the systematic and mutually verifiable study of the differences in the diaphragm muscle among young and old rats exposed to CMV. According to functional enrichment analyses, we found that CMV-induced molecular alterations and engaged pathways that differed distinctly between young and old rats. To our knowledge, this study is the first report of age-related changes in mRNA transcript profiling and Olink proteomics in the diaphragm in response to long-term mechanical ventilation using a unique experimental ICU model not limited by early mortality.

In the diaphragm of young rats, 5 days CMV resulted in decreases in the biological processes related to energy metabolism and production as indicated by the down-regulation of pathways like “carbon metabolism,” “citrate cycle (TCA cycle),” “pyruvate metabolism,” “glycolysis/gluconeogenesis,” “fatty acid metabolism,” “biosynthesis of amino acids,” and “oxidative phosphorylation.” The downregulation of metabolic pathways and “calcium signaling pathway” are suggested to reflect a physiological adaptation to the inactivation of the diaphragm by the post-synaptic blockade of neuromuscular transmission. The decreased expression of genes encoding structural sarcomeric proteins (see details in Supplementary Figure 2B) may contribute to the impaired regulation of diaphragm muscle contraction we have previously observed in response to long-term CMV in young rats [17].

In the diaphragm of old rats, five days CMV resulted in increases in the biological processes related to immune and inflammation responses as indicated by the up-regulation of pathways like “IL-17 signaling pathway,” “chemokine signaling pathway,” “cytokine-cytokine receptor interaction,” “TNF signaling pathway,” “NF-kappa B signaling pathway.” and “phagosome.” As shown in Fig. 4, Supplementary Figures 3 and 8, and Supplementary Table 1, an increased expression was observed in genes encoding CC chemokines (CCL2, CCL3, CCL5, CCL7, CCL17, CCL20, etc.), CXC chemokines (CXCL1, CXCL2, CXCL3, CXCL5, CXCL11, etc.), cytokines (IL-6, IL-1β, CSF2, etc.), and matrix metalloprotease (MMP9, MMP13, etc.). The change of some cytokines/chemokines at the gene level were reflected at the protein level, demonstrating an increase in CCL2, CCL3, CCL5, CCL12, CXCL1, CXCL2, CXCL3 (Fig. 4, Supplemental Figure 6 and 7). Thus, the age-related loss in diaphragm muscle function in response to long-term CMV, the delayed weaning process, increased mortality and dramatically increased health care costs in critically ill ICU patients are suggested to be closely linked to the cytokine storm and hyperinflammation induced by CMV.

It cannot be completely ruled out that other factors other than CMV induced the inflammatory response, such as 5 days exposure to post-synaptic blockade of neuromuscular transmission. We have previously shown that 5 days deep sedation and mechanical ventilation alone or in combination with neuromuscular blockade, corticosteroid hormone treatment, and sepsis separately or all combined have a strong negative effect on diaphragm muscle fiber function [24]. However, neuromuscular blockade, corticosteroid hormone treatment, or sepsis did not have an additional negative effect than in animals exposed to mechanical ventilation and deep sedation alone [24]. This is in sharp contrast to the effects on limb muscles where sepsis and corticosteroid treatment, but not neuromuscular blockade, had a strong negative effect on limb muscle fiber function [25]. Thus, the only common denominator resulting in the severely impaired diaphragm muscle fiber function was mechanical ventilation per se supporting the dominant role of CMV on the compromised diaphragm muscle function.

Old age is one of the factors most strongly associated with mortality in the general as well as in the specific COVID-19 ICU population. Mechanical ventilation is a life-saving intervention in the critically ill, but the current results suggest that mechanical ventilation should be restricted to as short duration as possible, especially the controlled mechanical ventilation. Other modes of mechanical ventilation such as negative pressure ventilation may be an alternative with less ventilator induced lung injury (VILI) than the most commonly used positive pressure ventilation used in ICUs [26], since VILI may be a critical factor in age-related lung injury and the subsequent release of factors with negative consequences for peripheral organs including respiratory muscles. Systemic release of factors in response to CMV were confirmed by plasma proteomic analysis, demonstrating elevated protein levels of a series of inflammatory factors and cytokines/chemokines, such as factors highly associated with “NF-kappa B signaling pathway,” “IL-17 signaling pathway,” “chemokine signaling pathway,” “cytokine-cytokine receptor interaction.” Notably, before CMV, higher basal levels of systemic inflammation were observed in old rats as well as a larger magnitude of the increase in systemic inflammation in old age after CMV. We suspect this phenomenon may be attributed to the effect of inflammaging.

Inflammaging is characterized by elevated basal levels of inflammation during aging, related to the accumulation of senescent cells and their secreted inflammatory molecules like cytokines and chemokines, a condition known as senescence-associated secretory phenotype (SASP) [2, 27]. Baseline inflammations need not be detrimental but preexisting inflammatory cells with SASP may participate in the inflammatory cascade and amplify the triggered inflammatory events [2]. The smoldering state of elevated inflammation may cause severe tissue damage due to toxicity of aged T lymphocytes [28], forwarded as an important factor causing hyperinflammation and lung injury in old patients with COVID-19 [2]. Results from this study, show that CMV triggers an inflammatory event amplified by inflammaging in old age.

The relatively small number of animals in each group represents a limitation of the study related to availability of old F344 BN hybrid rats together with and technically very demanding and labor intense experimental ICU model. The complexity of this demanding model is however outweighed by the advantage of studying the effects of the ICU condition for long durations as well as the strong age-specific effects demonstrated in the current study, although future studies in a larger population will generate more detailed information regarding specific pathways.

In conclusion, our study demonstrates significant age-related differences in the diaphragm response at gene and protein levels in response to long-term controlled mechanical ventilation of clinical importance for age-related weaning problems and mortality. In comparing enrichment pathways between young and old animals, results show that the diaphragm of old, but not young, rats was under a dramatic immune and inflammatory response during long-term CMV. This suggests that the use of mechanical  ventilation in old COVID-19 patients should be considered carefully, used for as short durations as possible (especially controlled mechanical ventilation). Other modes of life saving mechanical ventilation may be of importance causing less lung injury, such as negative pressure ventilation.

## Supplementary Information

**Additional file 1.** Supplemental Figure 1: Gene and protein signatures of the diaphragm in response to CMV. Supplemental Figure 2: Top GO terms and involved DEGs of the diaphragm in response to CMV in young rats. Supplemental Figure 3: Top GO terms and involved DEGs of the diaphragm in response to CMV in old rats. Supplemental Figure 4: “Oxidative phosphorylation” KEGG pathway and involved DEGs of the diaphragm in response to CMV in young rats. Supplemental Figure 5: “Calcium signaling pathway” and involved DEGs of the diaphragm in response to CMV in young rats. Supplemental Figure 6: “Cytokine-cytokine receptor interaction” KEGG pathway and involved DEGs and DEPs of the diaphragm in response to CMV in old rats. Supplemental Figure 7: “TNF signaling pathway” and involved DEGs and DEPs of the diaphragm in response to CMV in old rats. Supplemental Figure 8: “NF-kappa B signaling pathway” and involved DEGs and DEPs of the diaphragm in response to CMV in old rats.

**Additional file 2.** Supplemental Table 1: CMV-induced DEGs and DEPs in the diaphragm.

**Additional file 3.** Supplemental Table 2: CMV-induced DEPs in the plasma.

**Additional file 4.** Supplemental Table 3: Enriched GO terms in the diaphragm.

**Additional file 5.** Supplemental Table 4: Enriched KEGG pathways in the diaphragm.

**Additional file 6.** Supplemental Table 5: Enriched KEGG pathways in the plasma.

## Data Availability

The datasets used during the current study are available from the corresponding author on a reasonable request.

## Abbreviations

CCL2: C-C motif chemokine 2
CCL3: C-C motif chemokine 3
CCL5: C-C motif chemokine 5
CCL7: C-C motif chemokine 7
CCL17: C-C motif chemokine 17
CCL20: C-C motif chemokine 20
CXCL1: Growth-regulated alpha protein
CXCL2: C-X-C motif chemokine 2
CXCL3: C-X-C motif chemokine 3
CXCL5: C-X-C motif chemokine 5
CXCL9: C-X-C motif chemokine
Cpe: Carboxypeptidase E
Eda2r: Tumor necrosis factor receptor superfamily member 27
Tnfrsf12a: Tumor necrosis factor receptor superfamily member 12a
Itgb1bp2: Integrin beta-1-binding protein 2
Fst: Follistatin
Cyr61: Protein CYR61
Epo: Erythropoietin
IL-6: Interleukin-6
IL-1B: Interleukin-1 beta
CSF2: Granulocyte-macrophage colony-stimulating factor 2
MMP9: Matrix metallopeptidase 9
MMP13: Matrix metallopeptidase 13
CIM: Critical illness myopathy
VIDD: Ventilator-induced diaphragm dysfunction
BP: Biological process
MF: Molecular function
CC: Cellular component
Me: Metabolism
GIP: Genetic information processing
EIP: Environmental information procession
CP: Cellular processes
OS: Organismal system
HD: Human disease
DD: Drug development
SASP: Senescence-associated secretory phenotype
