# Supplementary material for: Multi-omics reveals age-related differences in the diaphragm response to mechanical ventilation: a pilot study
Source: Skelet Muscle. 2021 May 3;11:11. doi: 10.1186/s13395-021-00267-4 (PMC8089133; doi:10.1186/s13395-021-00267-4)
Supplement: Supplementary file 1 — Additional file 1. Supplemental Figure 1: Gene and protein signatures of the diaphragm in response to CMV. Supplemental Figure 2: Top GO terms and involved DEGs of the diaphragm in response to CMV in young rats. Supplemental Figure 3: Top GO terms and involved DEGs of the diaphragm in response to CMV in old rats. Supplemental Figure 4: “Oxidative phosphorylation” KEGG pathway and involved DEGs of the diaphragm in response to CMV in young rats. Supplemental Figure 5: “Calcium signaling pathway” and involved DEGs of the diaphragm in response to CMV in young rats. Supplemental Figure 6: “Cytokine-cytokine receptor interaction” KEGG pathway and involved DEGs and DEPs of the diaphragm in response to CMV in old rats. Supplemental Figure 7: “TNF signaling pathway” and involved DEGs and DEPs of the diaphragm in response to CMV in old rats. Supplemental Figure 8: “NF-kappa B signaling pathway” and involved DEGs and DEPs of the diaphragm in response to CMV in old rats. [file 13395_2021_267_MOESM1_ESM.docx]

**Multi-omics reveals age-related differences in the diaphragm response to**

**mechanical ventilation**

**Online Data Supplement**

Qiong Lyu, Ya Wen, Xiang Zhang, Alex B. Addinsall, Nicola Cacciani and Lars Larsson

**Supplemental Figure legends**

**Supplemental Figure 1. Gene and protein signatures of the diaphragm in response to CMV.** (**A**) Venn diagram of detected genes in each group; (**B**) Up- and down-regulated DEGs in the diaphragm in response to CMV of both young and old rats; Volcano plots of CMV-induced DEPs in the diaphragm of (**C**) young rats (**D**) old rats; Volcano plots of CMV-induced DEPs in the plasma of (**E**) young rats (**F**) old rats.

**Supplemental Figure 2. Top GO terms and involved DEGs of the diaphragm in response to CMV in young rats.** (**A**) top 3 GO terms in BP category; (**B**) top 3 GO terms in CC category. BP: Biological Process; CC: Cellular component. Fold change information of each DEG is indicated in colors from low (green) to high (red).

**Supplemental Figure 3. Top GO terms and involved DEGs of the diaphragm in response to CMV in old rats.** (**A**) top 5 GO terms in BP category; (**B**) top 1 GO terms in CC category. BP: Biological Process; CC: Cellular component. Fold change information of each DEG is indicated in colors from low (green) to high (red).

**Supplemental Figure 4. “Oxidative phosphorylation” KEGG pathway and involved DEGs of the diaphragm in response to CMV in young rats.** Fold change information of each DEG is indicated in color from low (green) to high (red).

**Supplemental Figure 5. “Calcium signaling pathway**” **and involved DEGs of the diaphragm in response to CMV in young rats.** Fold change information of each DEG is indicated in color from low (green) to high (red).

**Supplemental Figure 6. “Cytokine-cytokine receptor interaction” KEGG pathway and involved DEGs and DEPs of the diaphragm in response to CMV in old rats.** Ccl2, Ccl3, Cxcl1, Cxcl2, Cxcl3, and Edar2 were identified both as the CMV-induced DEGs and CMV-induced DEPs. Fold change information of each DEG is indicated in color from low (green) to high (red). Fold change information of each DEP is indicated in color from low (blue) to high (yellow).

**Supplemental Figure 7. “TNF signaling pathway” and involved DEGs and DEPs of the diaphragm in response to CMV in old rats.** Casp3, Ccl2, Cxcl1, Cxcl2, and Cxcl3 were identified both as the CMV-induced DEGs and CMV-induced DEPs. Fold change information of each DEG is indicated in color from low (green) to high (red). Fold change information of each DEP is indicated in color from low (blue) to high (yellow).

**Supplemental Figure 8. “NF-kappa B signaling pathway” and involved DEGs and DEPs of the diaphragm in response to CMV in old rats.** Edar2 and MIP-2 were identified both as the CMV-induced DEGs and CMV-induced DEPs. Fold change information of each DEG is indicated in color from low (green) to high (red). Fold change information of each DEP is indicated in color from low (blue) to high (yellow).

**Supplemental Figure 1**


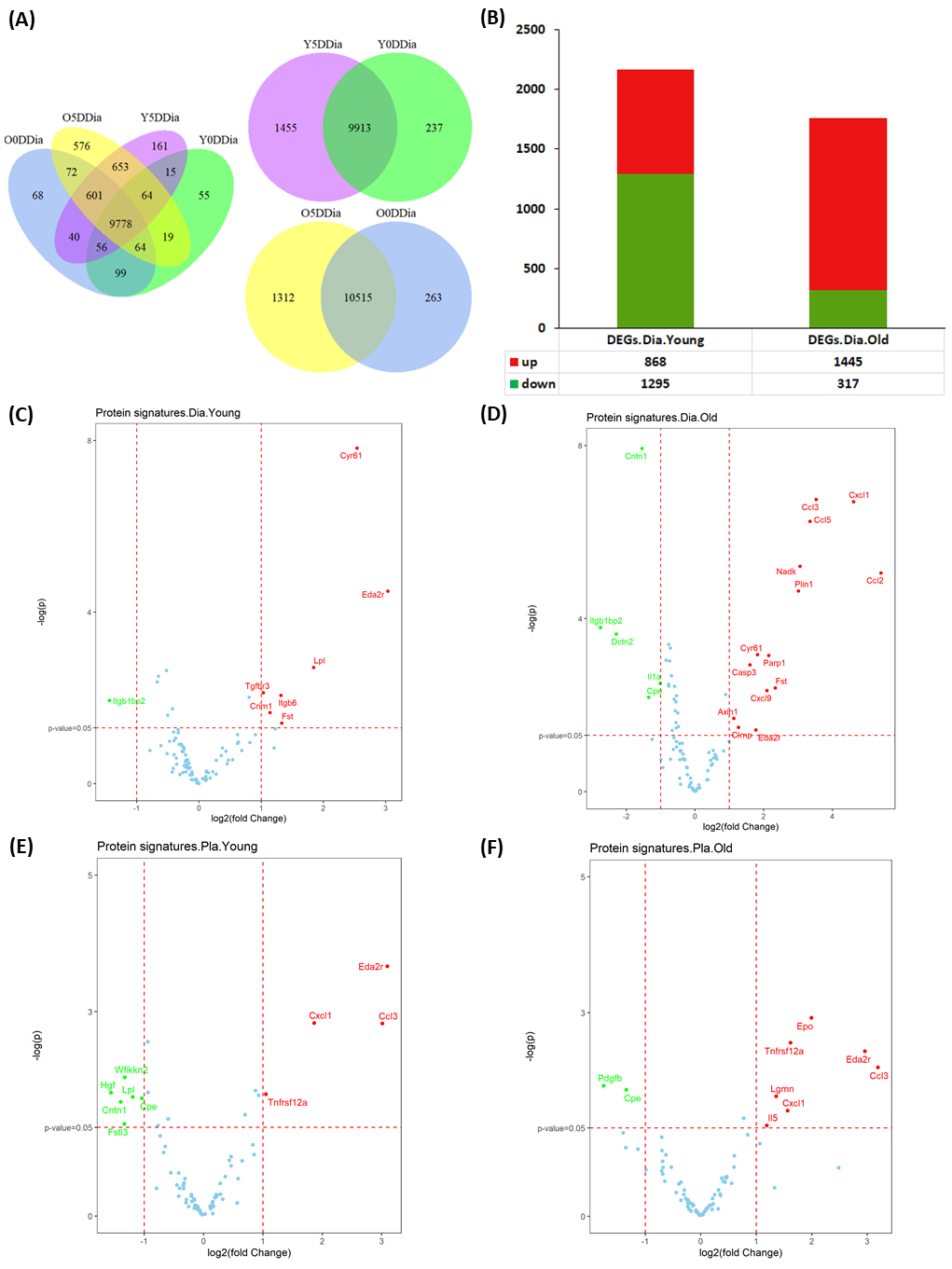


**Supplemental Figure 2**


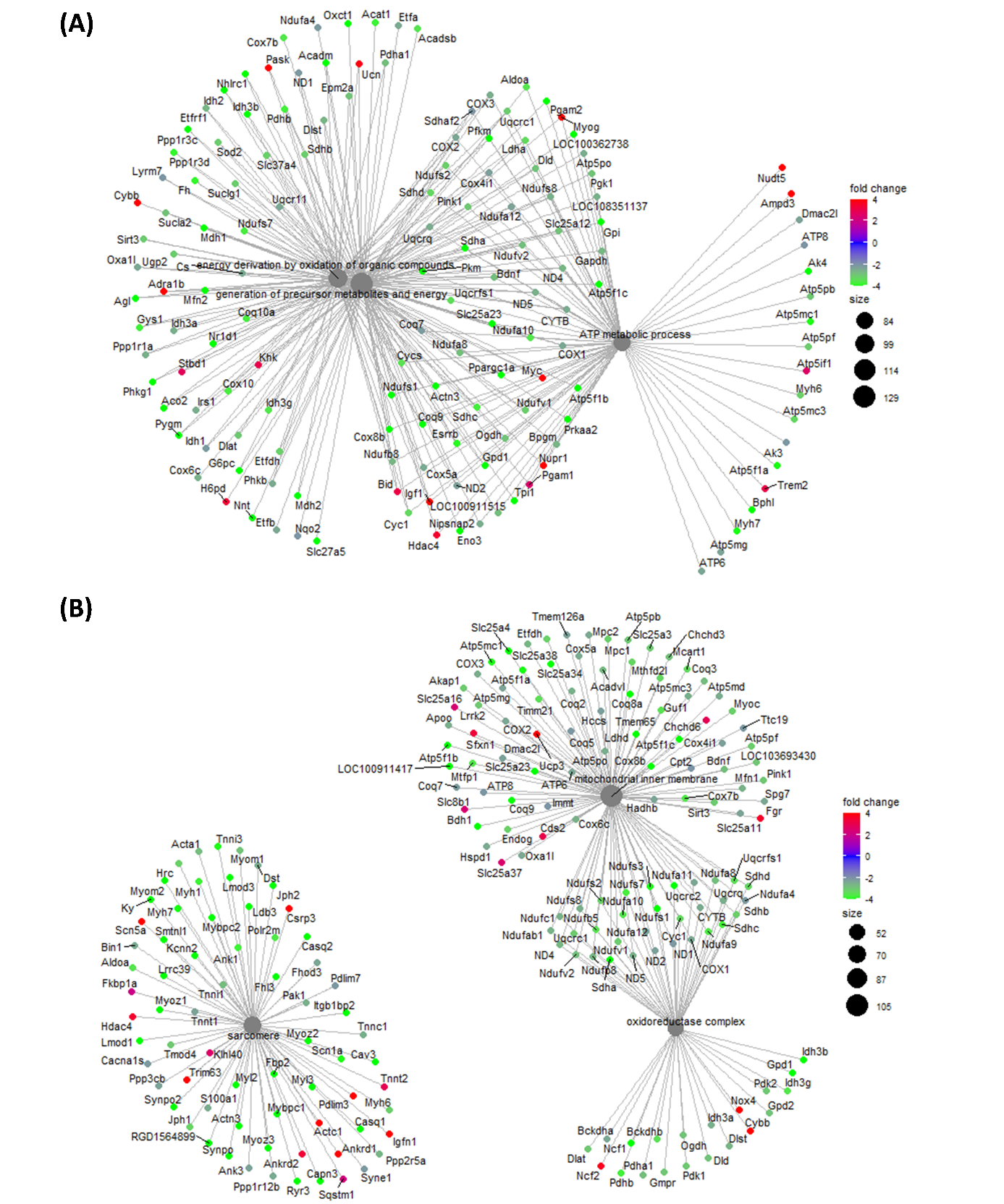


**Supplemental Figure 3**


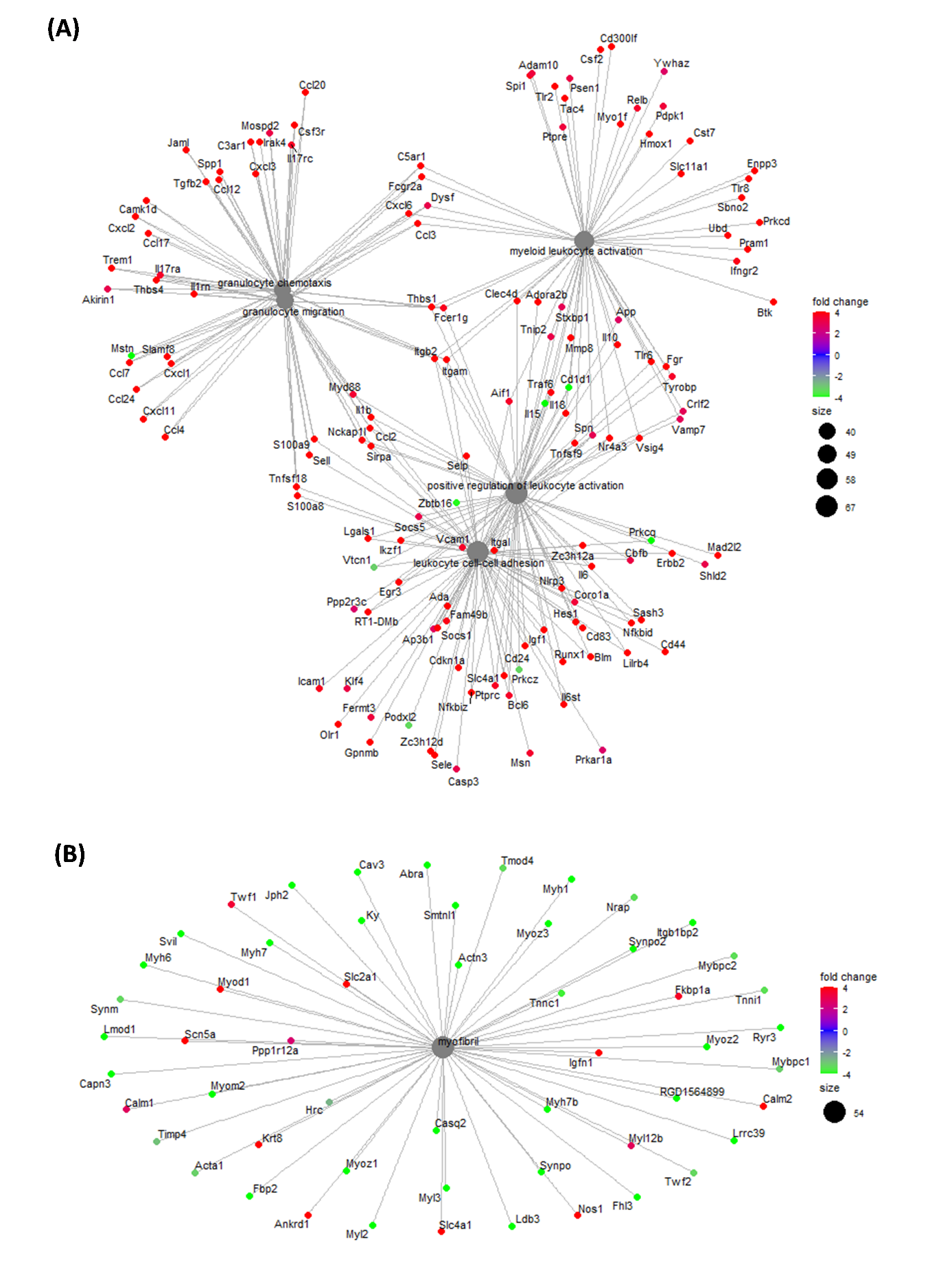


**Supplemental Figure 4**


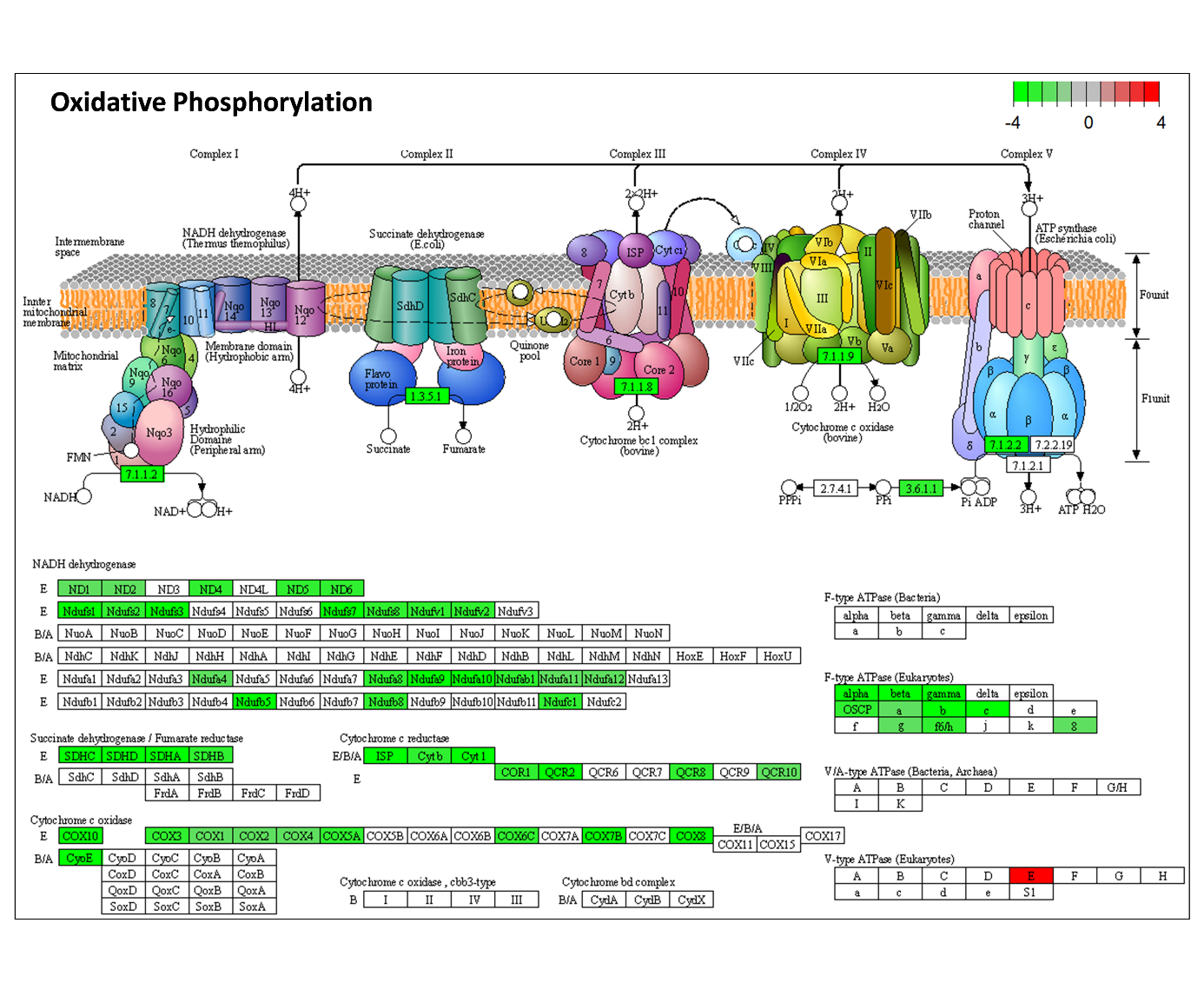


**Supplemental Figure 5**


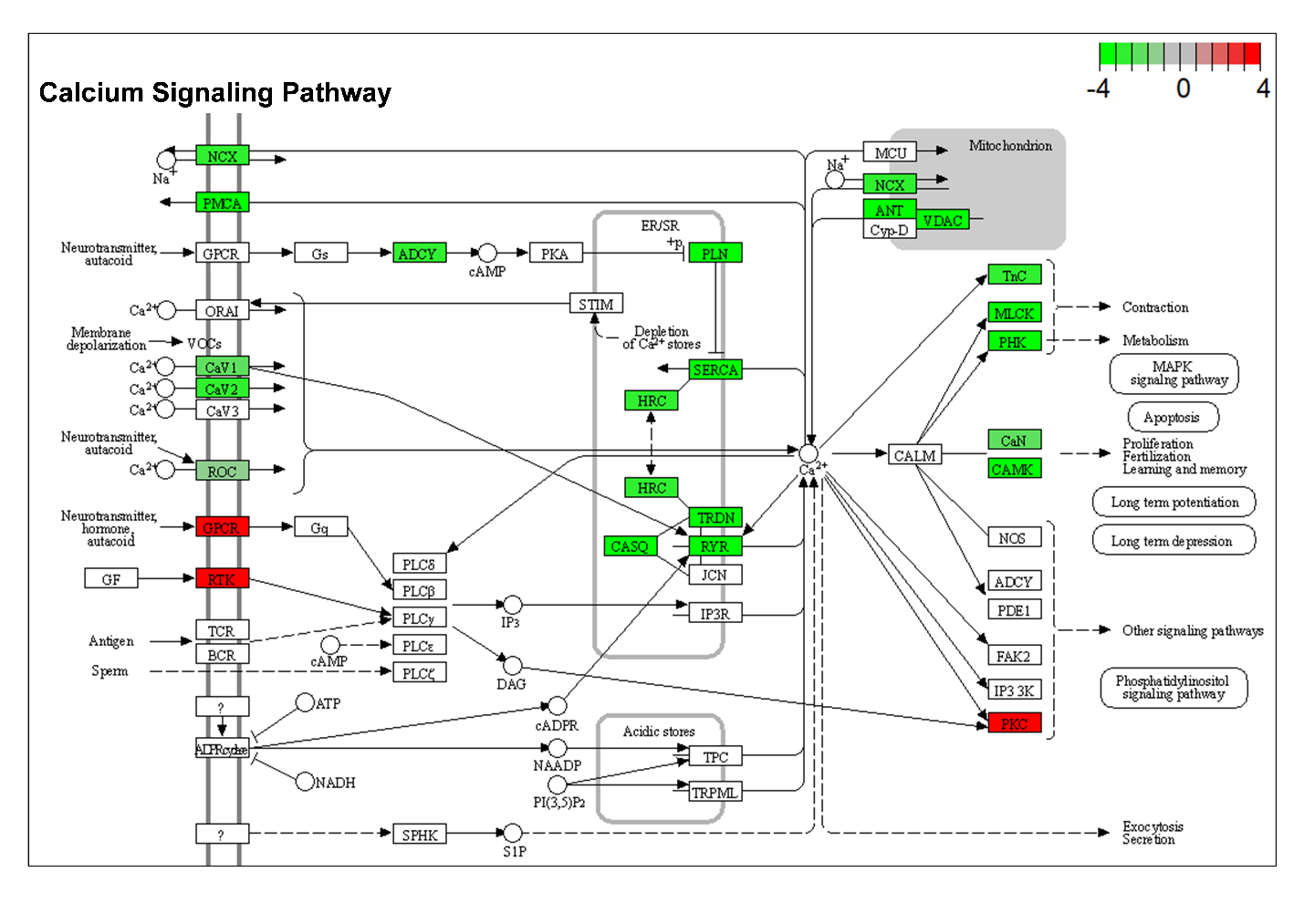


**Supplemental Figure 6**


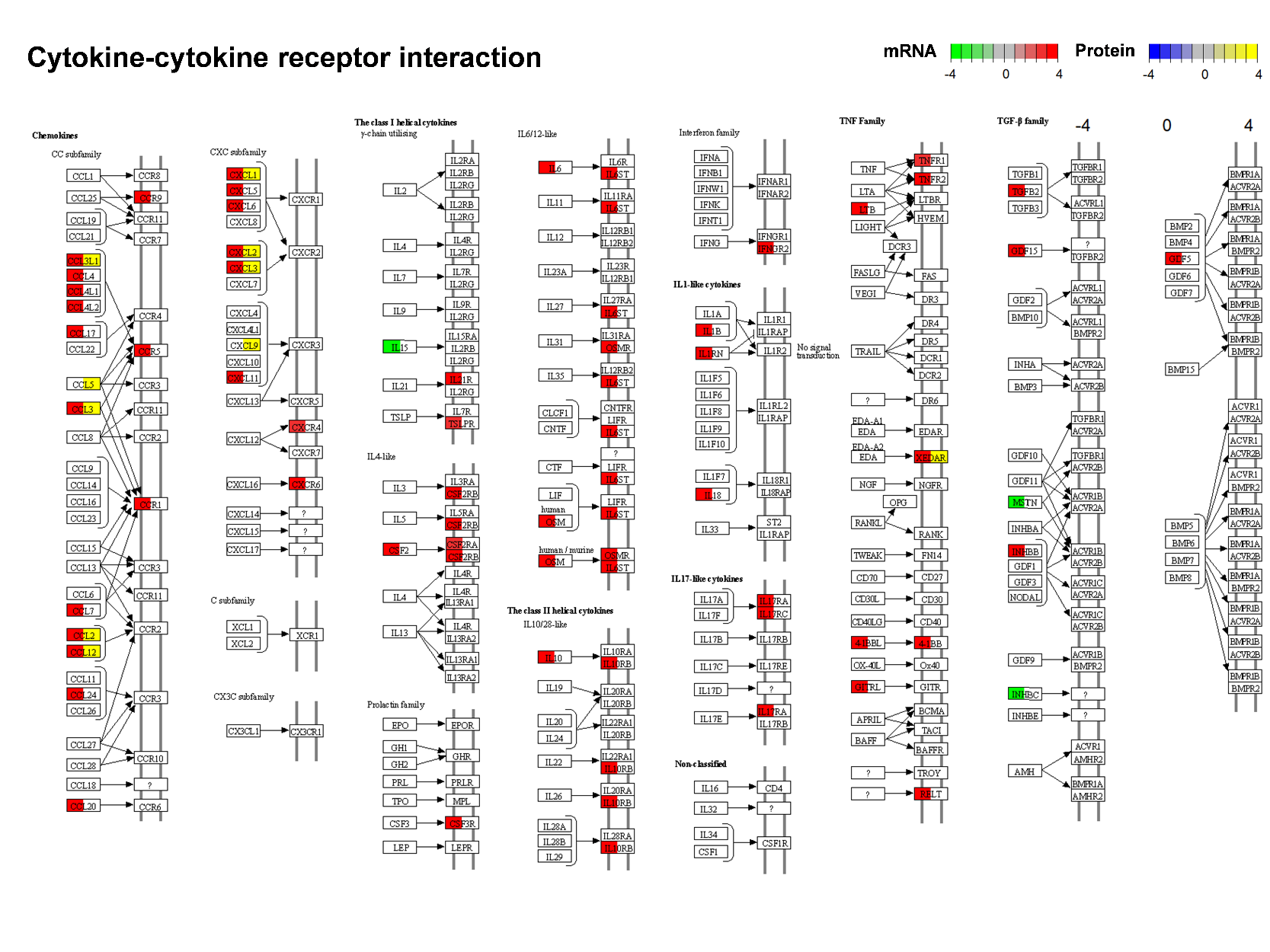


**Supplemental Figure 7**


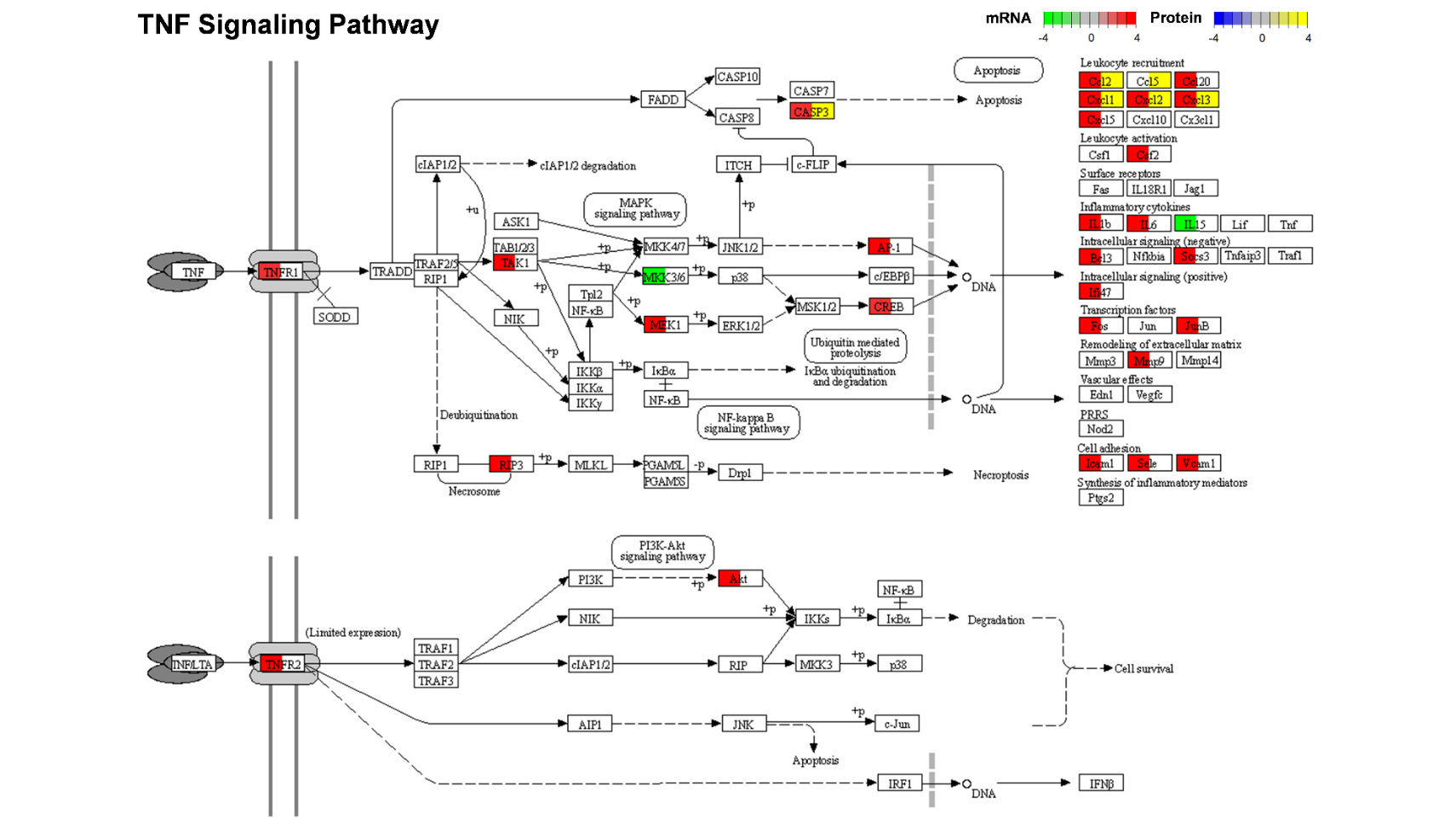


**Supplemental Figure 8**


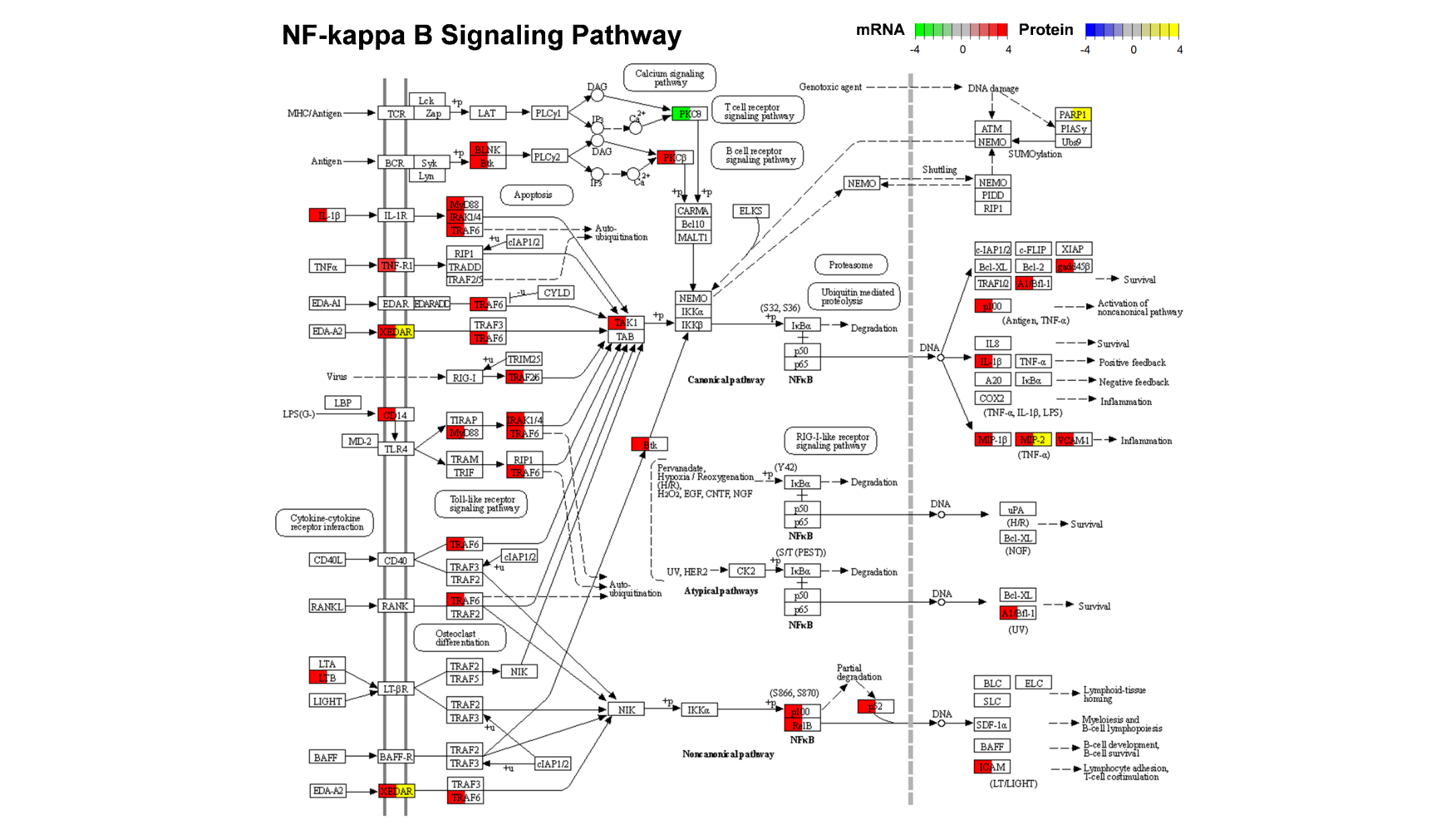


**Supplemental Table Description**

**Supplemental Table 1. CMV-induced DEGs and DEPs in the diaphragm.** CMV-induced DEGs in the diaphragm of **(A)** young rats and **(B)** old rats; CMV-induced DEPs in the diaphragm of **(C)** young rats and **(D)** old rats.

**Supplemental Table 2. CMV-induced DEPs in the plasma.** CMV-induced DEPs in the plasma of **(A)** young rats and **(B)** old rats**.**

**Supplemental Table 3. Enriched GO terms in the diaphragm.** GO terms enriched by CMV-induced DEGs in the diaphragm of **(A)** young rats and **(B)** old rats; GO terms enriched by CMV-induced DEPs in the diaphragm of **(C)** young rats and **(D)** old rats.

**Supplemental Table 4. Enriched KEGG pathways in the diaphragm.** KEGG pathways enriched by CMV-induced DEGs in the diaphragm of **(A)** young rats and **(B)** old rats; KEGG pathways enriched by CMV-induced DEPs in the diaphragm of **(C)** young rats and **(D)** old rats.

**Supplemental Table 5. Enriched KEGG pathways in the plasma.** KEGG pathways enriched by CMV-induced DEPs in the plasma of **(A)** young rats and **(B)** old rats.
